# Alkaloidal Variation in *Cissampelos Capensis* (Menispermaceae)

**DOI:** 10.3390/molecules16043001

**Published:** 2011-04-07

**Authors:** Helene de Wet, Fanie R. van Heerden, Ben-Erik van Wyk

**Affiliations:** 1 Department of Botany, University of Zululand, P/Bag X1001, Kwa-Dlangezwa 3880, South Africa; 2 School of Chemistry, University of KwaZulu-Natal, P/Bag X01, Scottsville 3209, Pietermaritzburg, South Africa; 3 Department of Botany and Plant Biotechnology, University of Johannesburg, P.O. Box524, Auckland Park 2006, Johannesburg, South Africa

**Keywords:** Menispermaceae, *Cissampelos capensis*, major alkaloids, medicinal uses, southern Africa

## Abstract

*Cissampelos capensis*, commonly known by the Afrikaans name “dawidjies” or “dawidjieswortel”, is the most important and best known medicinal plant of the family Menispermaceae used by the Khoisan and other rural people in the western region of South Africa. The main alkaloids in the leaves, stems and rhizomes were isolated and identified. Several of the main compounds were previously found in species of the related genus *Antizoma* and this similarity indicates that the two genera are closely related if not congeneric. Bulbocapnine (an aporphine alkaloid), dicentrine (an aporphine alkaloid) and salutaridine (a morphinane alkaloid) were the main alkaloids in the leaves, while bulbocapnine, cissacapine, cycleanine and insularine (the last three are bisbenzyltetrahydro-isoquinoline alkaloids) are the major compounds in the stems. The rhizome contains mostly bisbenzyltetrahydroisoquinoline alkaloids, with 12-*O*-methylcurine, cissacapine and cycleanine as the main ones. Alkaloids appear to be quite variable within different plant parts and different provenances, as confirmed by the difference in alkaloid patterns between coastal and inland forms of *Cissampelos capensis* (the morphinane alkaloid salutaridine, for example, is the major leaf alkaloid along the coast but is practically absent from the inland form of the species). The variety of alkaloids identified may contribute to the medicinal value of this species. The data on alkaloidal variation in the species has potential value and practical applications in chemotaxonomy, toxicology and pharmacognosy.

## 1. Introduction

In this paper the presence, identity and variation of the main alkaloids of *Cissampelos capensis* L.f. (Menispermaceae) are reported for the first time. Despite its importance in traditional medicine, almost nothing has yet been published on the alkaloids of this species. Accurate identification of the main alkaloids was considered to be an important first step to gain deeper insight into the value of the plant in traditional medicine. A further aim was to investigate possible geographical variation in the alkaloids found in different populations (provenances) as well as possible chemical differences between various plant parts, especially rhizomes – the part that is mainly used in traditional medicine – and leaves, which are reported to be toxic to cattle [1].

**Figure 1:**
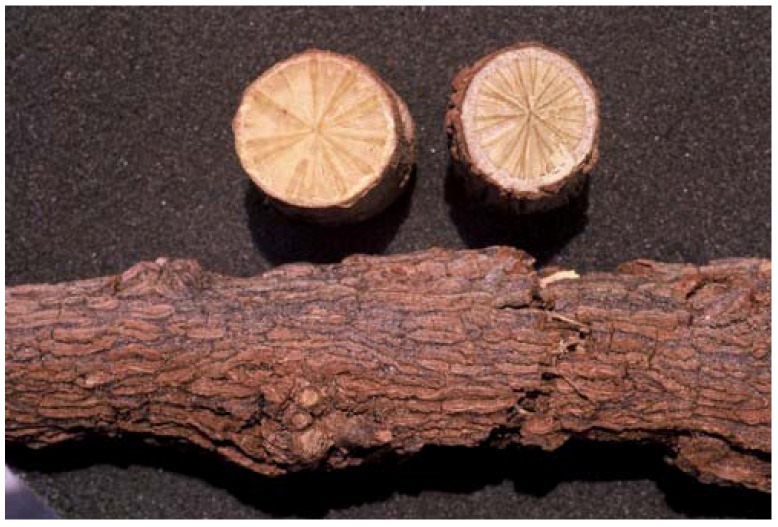
Rhizome of *Cissampelos capensis.*

The genus *Cissampelos* L. is one of seven genera of the Menispermaceae indigenous to southern Africa. In many regions of the world, members of the family are well known for their medicinal uses, which are associated with their rich diversity of isoquinoline alkaloids [2]. The genus is represented in southern Africa by four species: *C. capensis*, *C. hirta* Klotzsch, *C. mucronata* A. Rich. and *C. torulosa* E. Mey. ex Harv. [3]. *Cissampelos capensis* is the only endemic species in southern Africa and occurs in the winter rainfall region. The plant is a rambling shrub with thick, divergent branches and twining stems. Inland populations show xerophytic adaptations (small glaucous leaves) but along the coast the leaves tend to be larger and less glaucous [4,5,6]. *Cissampelos capensis* is of special significance in Khoisan ethnomedicine [3,7,8]. The rhizomes, known as “dawidjies” or “dawidjiewortel” (Figure 1) are widely used in traditional medicine as a blood purifier and a diuretic medicine [1]. 

It reduces fever and soothes pain and is also taken for diabetes, tuberculosis, stomach and skin cancers [7,8]. It also helps with pregnancy related and menstrual problems [9]. Leaf paste is used topically only – on snakebite wounds and syphilitic sores [1]. It is generally assumed that the activity of the rhizomes is due to alkaloids, but nothing has hitherto been published on the chemical compounds of *C. capensis* except for brief references to a few of the main compounds [4,5]. These were included in two reference books and were based on our own unpublished data. The first is a book on poisonous plants of South Africa [4] where the presence of glaziovine in leaves and insularine in rhizomes was merely stated but without any reference to supporting data. The second is a book on the medicinal plants of South Africa [5], where once again the presence of insularine, glaziovine and cissacapine was mentioned without any references or supporting data. There are as yet no scientific papers describing any alkaloids from *C. capensis*.

## 2. Results and Discussion

**Figure 2:**
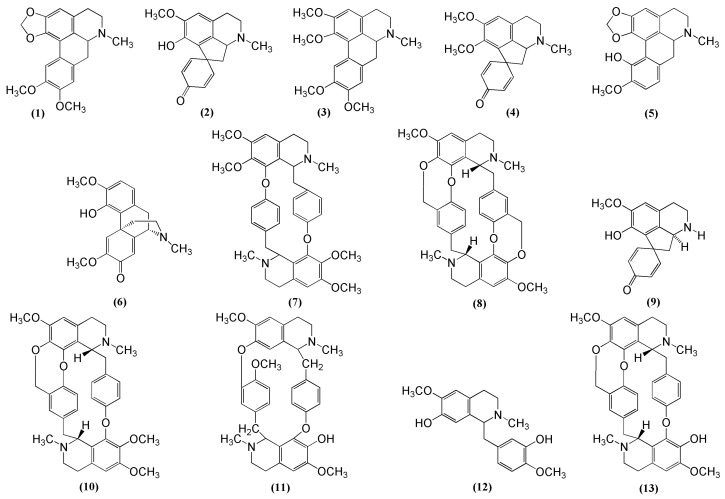
Chemical structures of the major alkaloids (**1-13**) isolated from *Cissampelos capensis*. dicentrine (**1**), glaziovine (**2**), lauroscholtzine (**3**), pronuciferine (**4**), bulbocapnine (**5**), salutaridine (**6**), cycleanine (**7**), cissacapine (**8**), crotsparine (**9**), insularine (**10**), 12-*O*-methylcurine (**11**), reticuline (**12**), insulanoline (**13**).

The crude alkaloid yields of *C. capensis* were very variable and ranged from 0.2 to 33.9 mg per gram dry weight (Table 1). The average yield of the crude alkaloid extracts from leaves was much higher than from stems and rhizomes, except for the high yield obtained from two bulk rhizome samples (yields of 33.9 and 6.9 mg per gram dry weight). The leaves of *C. capensis* yielded three major alkaloids, dicentrine (**1**), bulbocapnine (**5**), and salutaridine (**6**), with a high concentration of glaziovine (**2**) in only one sample (LS, plant 1b) and four minor alkaloids glaziovine (**2**), lauroscholtzine (**3**), cycleanine (**7**) and crotsparine (**9**) (Table 1 and Figure 2). 

**Table 1:** Distribution and yields of main alkaloids (%) as determined by HPLC in *Cissampelos capensis* leaves, stems and rhizomes. Alkaloids are arranged by their structural types (**A** aporphine, **P** proaporphine, **BB** bisbenzyltetrahydroisoquinoline, **B** benzyltetrahydro-isoquinoline, **M** morphinane) and numbered as in Figure 1. In column 1, a and b refer to different plants within the same population. (+) = trace amount; (–) = not detected.

Populations	Alkaloid yield	A	A	A	P	P	P	BB	B	BB	BB	BB	B	M
	(mg/g dry wt)	5	1	3	9	2	4	11	8	7	13	10	12	6
**Leaves^a^**														
CW, plant 4068-a	2.5	14	74	2	1	9	–	–	–	–	–	–	–	–
CW, plant 4068-b	19.0	9	78	4	1	+	–	–	–	+	–	–	–	8
LS, plant 1b	6.4	1	20	+	1	77	–	–	–	+	–	–	–	1
GR, plant 2a	3.4	3	96	–	–	–	–	–	–	1	–	–	–	–
GR, plant 2b	3.2	1	89	2	–	–	–	–	–	8	–	–	–	–
SB, plant 3a	9.6	21	44	6	+	+	–	–	–	2	–	–	–	27
SB, plant 3b	10.5	10	35	8	+	–	–	–	–	+	–	–	–	47
MB, plant 4a	15.2	53	+	6	+	+	–	–	–	7	–	–	–	33
MB, plant 4b	6.1	33	+	9	1	+	–	–	–	4	–	–	–	53
SF, plant 5a	8.4	43	13	5	1	1	–	–	–	6	–	–	–	31
SF, plant 5b	4.7	5	47	6	3	1	–	–	–	1	–	–	–	37
**Stems**														
CW, plant 4068	0.2	3	16	–	–	–	–	–	58	–	–	23	–	–
LS, plant 1b	0.6	56	–	–	–	–	–	–	–	18	–	2	15	–
GR, plant 2a	2.3	6	–	–	–	–	–	–	13	43	1	7	1	–
GR, plant 2b	0.5	3	–	–	–	–	–	–	43	23	2	20	–	–
SB, plant 3b	1.4	18	17	–	–	–	–	21	30	1	3	9	–	–
MB, plant 4a	0.7	7	–	–	–	–	–	8	45	11	3	15	–	–
SF, plant 5a	0.7	–	5	–	–	–	–	–	38	24	14	19	–	–
**Rhizomes**														
LS, bulk-I sample	33.9	+	50	–	–	–	–	+	46	–	–	–	–	–
SB, bulk-S sample	6.9	1	17	–	–	2	–	1	–	14	–	1	+	–
LS, plant 1a	1.6	–	–	–	–	–	1	54	–	44	–	1	–	–
LS, plant 1b	1.0	2	–	–	–	–	3	27	–	67	–	1	–	–
GR, plant 2a	1.1	2	–	–	–	–	–	15	–	82	–	1	–	–
GR, plant 2b	1.1	1	–	–	–	–	3	32	–	63	–	1	–	–
SB, plant 3a	1.3	6	–	–	–	–	–	–	24	43	–	6	–	–
SB, plant 3b	2.3	2	–	–	–	–	–	64	8	22	–	2	–	–
MB, plant 4a	0.9	–	–	–	–	–	–	48	23	27	–	2	–	–
MB, plant 4b	1.4	2	–	–	–	–	–	46	16	24	–	1	–	–
SF, plant 5a	0.5	–	–	–	–	–	–	–	–	+	–	+	–	–
SF, plant 5b	1.9	–	–	–	–	–	–	–	–	77	–	12	–	–

A distinct difference was found between the inland and coastal populations. Leaves from the coastal populations (SB, plant 3; MB, plant 4 and SF, plant 5) [Table 2] had high concentrations of salutaridine (**6**), in contrast to the absence or low concentration of **6** in the inland populations (CW plant 4068, LS plant 1 and GR plant 2) [Table 1]. Eight alkaloids were identified in the stems, with bulbocapnine (**5**), cycleanine (**7**), cissacapine (**8**) and insularine (**10**) the most prominent ones (Table 1 and Figure 1). Cissacapine (**8**) is a new alkaloid with antiplasmodial activity extracted from rhizomes of *C. capensis* [4,5], the structural elucidation of which will be published elsewhere (Van Heerden FR *et al.*, unpublished results). The four minor alkaloids identified in the stems of only certain populations were dicentrine (**1**), 12-*O*-methylcurine (**11**), reticuline (**12**) and insulanoline (**13**). In contrast to the stem, cycleanine (**7**) and 12-*O*-methylcurine (**11**) were identified in the rhizomes as the major alkaloids. Relatively high concentrations of cissacapine (**8**) were identified in only two of the six populations and glaziovine (**2**), pronuciferine (**4**), bulbocapnine (**5**) and insularine (**10**) were detected in only some populations (and in low percentage yields). The morphinane alkaloid salutaridine (**6**), one of the major alkaloids in the leaves of the coastal form, was absent from the stems and the rhizomes.

Further confirmation and analytical verification was achieved by comparing the identities of the isolated compounds with the HPLC and TLC results of their respective crude extracts. In most cases these were found to be in close agreement. A notable exception was the presence of large amounts of dicentrine (**1**) and cissacapine (**8**) in the bulk samples, but the apparent absence of these compounds in analytical samples from the same populations. This seems to be due to large plant to plant variation. Note, for example, the presence of 12-*O*-methylcurine (**11**) as main compound (64%) in one plant from Still Bay, its apparent absence from another plant of the same population and the small yield (1%) of this compound in the corresponding bulk sample.

Table 1 shows considerable variation in the alkaloid distributions and yields among different populations, different plants in a population and different plant parts. There is no obvious explanation for the extreme quantitative variation, which may result from different developmental stages of the plants, genetic differences between individual plants and populations, as well as seasonal changes. Qualitative variation can possibly be explained by particular biochemical pathways in alkaloid synthesis being switched on or off as a result of environmental and/or genetic influences.

## 3. Experimental

### 3.1. General

NMR spectra (^1^H and ^13^C) were recorded on either a Varian Gemini 300 MHz or a Varian Inova 300 MHz spectrometer in CDCl_3_ using TMS as internal standard. Apart from ^1^H and ^13^C, COSY, NOESY, DEPT, HMQC and HMBC experiments were performed to elucidate the structures of compounds. EI-MS was recorded on a Shimadzu GCMS QP2010 apparatus. Optical rotations were measured on a JASCO DIP 370 digital polarimeter. Column chromatography was performed using silica gel 60 (230-400 mesh) and cyclohexane-chloroform-diethylamine (50:40:10) as the eluent. Analytical TLC of compounds or extracts was performed on Silica Gel 60 F_245_ Merck plates, using the same eluent system as for column chromatography. HPLC analyses were done on a Shimadzu 10A system with a binary gradient system and photodiode array detector, using a Waters Xterra RP C18 column and the following linear gradient solvent system: 0-90% acetonitrile in a 10 mM ammonium acetates solution (pH 9.5) over 50 min.

### 3.2. Plant Materials

Bulk material and material for analytical studies were collected from six different localities in South Africa. Voucher specimen details are listed in Table 2.

### 3.3. Extraction, Purification and Identification of Alkaloids

Plant material was separated into the different parts (leaves, stems and rhizomes), air-dried at maximum 40 ºC and then finely ground. Alkaloids were extracted at room temperature using 0.05 M H_2_SO_4_. After 1 hour, the pH was adjusted to 7 with 25% ammonia solution and the alkaloids extracted by phase separation with CH_2_Cl_2_ in a separating funnel.

**Table 2:** Voucher specimens of the material of *Cissampelos capensis* used for alkaloid isolation and identification. The specimens are kept in The Herbarium, Botany Department, University of Zululand, KwaZulu-Natal, South Africa (acronym UZ) and The Herbarium, Department of Botany and Plant Biotechnology, University of Johannesburg, Gauteng, South Africa (acronym JRAU).

Voucher specimens	Locality in South Africa, with quarter degree grid reference
De Wet 2 (UZ) De Wet 2 (UZ); SF, plant 5	Sedgefield [3422 BB (Mossel Bay)]
De Wet 3 (UZ) De Wet 3 (UZ); SB, plant 3; SB, bulk-S	Still Bay [3421 AD (Riversdale)]
De Wet 4 (UZ) De Wet 4 (UZ); MB, plant 4	Mossel Bay [3422 AA (Mossel Bay)]
De Wet 10 (UZ); LS, plant 1; LS, bulk-I	15 km east of Ladismith [3321 AD (Ladismith)]
De Wet 11 (UZ); GR, plant 2	Clifton farm, Graaff-Reinet [3224 BC (Graaff-Reinet)]
Van Wyk & De Wet 4068 (JRAU); CW, plant 4068	Boskloof farm, Clanwilliam [3218 BB(Clanwilliam)]

The organic extract was filtered through a glass column, packed with a coarse celite-577. Removal of the solvent under reduced pressure yielded the crude alkaloidal extracts. Alkaloids from the bulk rhizome and leaf extracts were isolated by column chromatography. Leaves (0.24 kg dry material) collected from the inland yielded pure samples of dicentrine (**1**; 8 mg) [see Figure 1 for all structures **1-13**], glaziovine (**2**; 30 mg), lauroscholtzine (**3**; 7 mg) and pronuciferine (**4**; 2.5 mg). Leaves (0.17 kg dry material) collected from the coast yielded pure samples of bulbocapnine (**5**; 30 mg) and salutaridine (**6**; 53 mg). Glaziovine (**2**) was also isolated from *Antizoma angustifolia* [10] and bulbocapnine (**5**) from *A. miersiana* [11].

*Dicentrine* (**1**). ^1^H-NMR (CDCl_3_) δ: 7.65 (1H, s, H-11), 6.76 (1H, s, H-8), 6.49 (1H, s, H-3), 6.05 (1H, d, *J* = 1.5 Hz, OCHO), 5.91 (1H, d, *J* = 1.5 Hz, OCH_2_O), 3.90 (3H, s, OCH_3_), 3.89 (3H, s, OCH_3_), 3.09 (4H, m), 2.62 (3H, m), 2.53 (3H, s, *N*-CH_3_). ^13^C-NMR (CDCl_3_) δ: 148.1 (C-9), 147.6 (C-10), 146.5 (C-2), 141.7 (C-1), 128.2 (C-7a), 126.5 (C-3a), 126.2 (C-1b), 123.4 (C-11a), 116.5 (C-1a), 111.2 (C-11), 110.4 (C-8), 106.7 (C-3), 100.6 (OCH_2_O), 62.4 (C-6a), 56.1 (OMe), 55.9 (OMe), 53.6 (C-5), 43.9 (N-Me), 34.2 (C-7), 29.2 (C-4). MS *m/z*: 39 (M^+^, 2), 178 (24), 97 (23), 85 (22), 83 (33), 81 (37), 71 (38), 70 (21), 69 (93), 67 (24), 60 (24), 59 (44), 57 (84), 56 (28), 55 (80), 45 (33), 44 (30), 43 (100), 41 (97).

*Lauroscholtzine* (**3**). ^1^H-NMR (CDCl_3_) δ: 7.90 (1H, s), 6.77 (1H, s), 6.63 (1H, s), 3.92 (3H, s, OMe), 3.89 (3H, s, OMe), 3.58 (3H, s, OMe), 2.52 (3H, s, NMe). MS *m/z*: 341 (M^+^, 23), 340 (25), 335 (20), 168 (82), 147 (44), 119 (22), 97 (30), 83 (22), 71 (25), 69 (100), 57 (50), 55 (40), 44 (53), 43 (55), 41 (44).

*Pronuciferine* (**4**). ^1^H-NMR (CDCl_3_) δ: 7.02 (1H, dd, *J* = 10.2 and 2.7 Hz, H-8), 6.86 (1H, dd, *J* = 9.9 and 2.7 Hz, H-12), 6.61 (1H, s, H-3), 6.38 (1H, dd, *J* = 9.9 and 2.0 Hz, H-11), 6.27 (1H, dd, *J* = 10.2 and 2.1 Hz, H-9), 3.78 (3H, s, 2-OMe), 3.57 (3H, s, 1-OMe), 2.34 (3H, s, NMe). ^13^C-NMR (CDCl_3_) δ: 186.1 (C-10), 153.5 (C-12), 153.3 (C-1 or 2), 150.0 (C-8), 134.3 (C-3a), 132.7 (C-7c), 128.2 (C-9), 127.7 (C-7b), 127.4 (C-11), 111.7 (C-3), 65.7 (C-6a), 61.1 (OMe), 56.3 (OMe), 54.9 (C-5), 51.2 (C-7a), 47.5 (C-7), 43.6 (N-Me), 27.5 (C-4). MS *m/z*: 312 (22), 311 (M^+^, 16), 335 (20), 310 (20), 169 (77), 147 (41), 119 (23), 97 (26), 83 (24), 71 (24), 69 (100), 59 (28), 57 (40), 55 (37), 44 (51), 43 (53), 42 (23), 41 (34).

*Salutaridine* (**6**). ^1^H-NMR (CDCl_3_) δ: 7.51 (1H, s, H-5), 6.71 (1H, d, *J* = 8.1 Hz, H-2), 6.62 (1H, br, d, *J* = 8.4 Hz, H-1), 6.29 (1H, s, H-8), 3.85 (3H, s, OCH_3_), 3.72 (3H, s, OCH_3_), 3.65 (1H, d, *J* = 4.8 Hz, H-9), 3.29 (1H, d, *J* = 17.7 Hz, H-10α), 2.94 (1H, ddd, *J* = 17.7, 5.7 and 1.2 Hz, H-10β), 2.57 (1H, ddd, *J* = 12.6, 4.8 and 1.8 Hz, H-14), 2.43 (1H, td, *J* = 12. and 3.3 Hz, H-14), 2.41 (3H, s, NMe), 2.33 (1H, ddd, *J* = 12.6, 3.0 and 1.8 Hz, H-15), 1.73 (1H, td, *J* = 12.0 and 4.5 Hz, H-15).^13^C-NMR (CDCl_3_) δ: 181.3 (C-7), 161.5 (C-14), 150.8 (C-6), 145.2 (C-3), 143.2 (C-4), 129.6 (C-11), 123.8 (C-12), 122.1 (C-8), 120.3 (C-5), 118.7 (C-1), 109.4 (C-2), 61.0 (C-9), 56.3 (OMe), 54.8 (OMe), 47.0 (C-16), 43.7 (C-13), 41.7 (NMe), 37.7 (C-15), 32.6 (C-10). MS *m/z*: 328 (22), 327 (M^+^, 100), 327 (83), 312 (44), 299 (73), 285 (25), 284 (100), 268 (27), 256 (23), 242 (30), 226 (23), 169 (61), 147 (28), 97 (24), 85 (32), 83 (59), 70 (21), 69 (64), 57 (30), 55 (33), 44 (59), 43 (37), 42 (54), 41 (26).

The rhizome (0.57 kg dry material) sample collected from the coast yielded pure sample of cycleanine (**7**; 31 mg), which was also isolated from *Antizoma miersiana* [11]. In the analytical samples (0.5 g dry material), the identified compounds were confirmed by comparison of R_t_ and UV spectra (HPLC-DAD) with reference compounds. Cissacapine (**8**), crotsparine (**9**) and insularine (**10**) were isolated from *Antizoma angustifolia* [10]. 12-*O*-methylcurine (**11**) and reticuline (**12**) were isolated from *Cissampelos hirta* and insulanoline (**13**) from *A. miersiana* [11].

## 4. Conclusions

The extreme variation in alkaloids should be taken into account if the plant is collected for medicinal purposes (a notable example is the almost complete absent of salutaridine (**6**) in the leaves of plants from inland localities). Traditional healers are often not aware that the same species at different localities are chemically different and therefore perhaps also different in its biological activity and potential toxicity. The fact that some of the leaf alkaloids of *C. capensis* are known to be toxic to animals indicate that the alkaloidal data may be of practical value in forensic studies in cases of human and animal poisoning. Most of the medicinal uses of this species can probably be attributed to its rich diversity of alkaloids, especially the predominance of bisbenzyltetrahydroisoquinoline alkaloids in the rhizome (the plant organ that is mostly used in traditional medicine). The three major alkaloids in the leaves are known to be biologically active. Bulbocapnine (**5**) has antimicrobial activity [12], dicentrine (**1**) has antibacterial and antifungal activity [13] while salutaridine (**6**) has anti-inflammatory activity [12]. These activities may explain the use of leaf poultices in treating wounds and syphilitic sores. Likewise, the biological activity of cycleanine (**7**) could possibly explain the medicinal uses of the rhizome. This bisbenzyltetrahydroisoquinoline alkaloid is known for its analgesic, muscle relaxant and anti-inflammatory effects [14] and also for its anti-carcinogenic activity [15].

In order to use alkaloids for chemotaxonomic purposes it is important to take into account the plant part used, the different stages of development and genetic variation of plants, the time of the year and the geographical distribution. From a chemotaxonomic point of view, the qualitative and quantitative patterns of alkaloids in *C. capensis* appear to be quite similar to those found in the two species of the southern African genus *Antizoma* Miers [10,11]. *Cissampelos capensis* agrees more closely with *Antizoma miersiana* in the presence of dicentrine (**1**) and bulbocapnine (**5**) as main compounds in the leaves, stems and rhizomes; *A. angustifolia* has crotsparine (**9**) as major constituent in leaves, stems and rhizomes, while this compound has so far only been detected in the leaves of *C. capensis* and *A. miersiana* but not in their stems or rhizomes. With the exception of cycleaneonine (present as minor alkaloid in the rhizomes of *A. miersiana* [11]), all the alkaloids found thus far in the genus *Antizoma* are also present in *C. capensis*. The data therefore supports the idea (based on morphological similarities) that the two genera are closely related and that *Antizoma* should perhaps be subsumed under *Cissampelos*. 
